# Elevated Coefficient of Variation in Total Fecal Bile Acids Precedes Diagnosis of Necrotizing Enterocolitis

**DOI:** 10.1038/s41598-019-57178-0

**Published:** 2020-01-14

**Authors:** Shannon Knapp, Allysa Kehring, Jennifer Stepp, Christine M. Calton, Sheila M. Gephart, Sruti Bandlamuri, Kate E. Boyle, Grey I. Dietz, Haeley Johnson, Ryan E. Romo, Mackenzie Spencer, Alan D. Bedrick, Melissa D. Halpern

**Affiliations:** 1 Statistics Consulting Lab, Bio5 Institute, University of Arizona Tucson, Tucson, AZ 85721 USA; 2 University of Arizona College of Medicine, 1501 N Campbell Ave, Tucson, AZ 85724 USA; 3 University of Arizona, Department of Pediatrics and Steele Children’s Research Center, PO Box 245073, Tucson, AZ 85724 USA; 4 University of Arizona College of Nursing, PO Box 210203, Tucson, AZ 85721 USA; 5 University of Arizona College of Medicine, 475 N. 5th Street, Phoenix, AZ 85004 USA

**Keywords:** Predictive markers, Infant necrotizing enterocolitis

## Abstract

Accumulation of bile acids (BAs) may mediate development of necrotizing enterocolitis (NEC). Serial fecal samples were collected from premature infants with birth weight (BW) ≤ 1800 g, estimated gestational age (EGA) ≤ 32 weeks, and <30 days old prior to initiation of enteral feeding. Nine infants that developed Bell’s Stage ≥ II NEC were matched with control infants based on BW, EGA, day of life (DOL) enteral feeding was initiated and DOL of the first sample. From each subject, five samples matched by DOL collected were analyzed for BA levels and composition. Fifteen individual BA species were measured via LC-MS/MS and total BA levels were measured using the Diazyme Total Bile Acid Assay kit. No statistically significant differences in composition were observed between control and NEC at the level of individual species (p = 0.1133) or grouped BAs (p = 0.0742). However, there was a statistically significant difference (p = 0.000012) in the mean coefficient of variation (CV) between the two groups with infants developing NEC having more than four-fold higher mean CV than controls. Importantly, these variations occurred prior to NEC diagnosis. These data suggest fluctuations in total fecal BA levels could provide the basis for the first predictive clinical test for NEC.

## Introduction

Necrotizing enterocolitis (NEC), a hemorrhagic inflammatory necrosis that mainly affects the distal ileum and colon^1^, is the most commonly diagnosed gastrointestinal emergency of premature infants^2,3^. Mortality rates range from 20–40 percent^2,4–6^ and survivors encounter hospital stays on average of 11 days longer than infants born at similar estimated gestational age that did not develop NEC^7^. Disease-associate costs of surgical NEC can extend beyond initial diagnosis, including development of short bowel syndrome and increased risk of neurodevelopmental morbidity^8,9^. While prematurity^10,11^, enteral feeding^12^ and intestinal bacterial colonization^13,14^ are recognized as major risk factors, definitive mechanisms of NEC pathophysiology have yet to be established and treatments remain primarily supportive. Methods that reliably predict which preterm infants are likely to develop NEC would greatly enhance clinical practice.

Bile acids (BAs) emulsify, absorb and transport fats and sterols in the intestine and liver. The primary BAs—cholic acid (CA) and chenodeoxycholic acid (CDCA)—are synthesized in hepatocytes, conjugated with either glycine or taurine, and then transported from the liver to the gall bladder for storage. After secretion into the intestine, BAs undergo bacterial dehydroxylation and conversion to their secondary forms—deoxycholic acid (DCA), lithocholic acid (LCA) and ursodeoxycholic acid (UDCA). Bacterial deconjugation of BAs occurs in the colon, and most are recirculated to the liver via the portal vein^15^. Alterations of these highly regulated processes may result in accumulation of cytotoxic, hydrophobic BAs (e.g., LCA, DCA, and CDCA) within enterocytes, with subsequent damage to the intestinal epithelium^16,17^.

We were the first to show accumulation of ileal BAs is critical to experimental NEC pathophysiology^18^, the mechanisms by which BAs accumulate in ileal enterocytes^19^, active transport of ileal BAs is required to decrease protective responses^20^, and the concomitant dysregulation of hepatic BA transporters^21^ during development of NEC. This novel paradigm encompasses major disease-associated findings and risk factors: 1) the more premature an infant, the more likely they will develop NEC^10,11^. Many of the key processes of BA homeostasis are not fully developed in neonates, but reach maturity at weaning^22–27^. 2) Formula-fed preemies are 6–10 times more likely to develop NEC^12^ and have higher fecal BA levels than breast-fed preemies^28^. Further, formula feeding is required to develop experimental NEC^29,30^. 3) The majority of BA reclamation occurs in the ileum and colon, the primary sites of NEC injury^31^, and ileal BA levels increase 24-hours before inflammation or histological damage in experimental NEC^32^. 4) Bacterial colonization is required for both development of NEC and formation of secondary BAs. While no specific pathogen has been conclusively associated with NEC^33–43^, disease cannot be developed in germ free conditions^13,14^ and pneumatosis intestinalis, the most pathognomonic radiological finding of NEC, is thought to be caused by bacterial overgrowth^44^. Bacteria are also required for formation of more toxic, hydrophobic BAs^45–47^, which are higher in both experimental^18^ and human NEC^48^.

To further characterize how BAs are dysregulated in human NEC, we prospectively collected fecal samples from premature infants and determined BA levels and composition from matched-pairs diagnosed with NEC versus those without NEC. Contrary to a previous study^48^, we found no statistically significant differences in BA composition between control and NEC. However, there was a statistically significant difference (p = 0.000012) in the mean coefficient of variation (CV) in the two groups with infants developing NEC having more than four-fold higher mean CV than controls. Importantly, these differences occurred prior to NEC diagnosis. These data strongly suggest the potential for utilization of total BA variability as the first predictive test for this devastating disorder.

## Results

### Matched pairs used for analysis

One hundred-sixty-nine infants were enrolled in the study and 12 developed NEC. Our final dataset was composed of nine NEC subject pairs matched to control subjects based on estimated gestational age (EGA), birth weight (BW), day of life (DOL) of initiation of enteral feeding and the DOL the first sample was collected. Five samples from each subject pair were matched based on DOL. All NEC subject samples measured were collected prior to NEC diagnosis and all subjects received antibiotics in the first week of life. Table 1 shows the characteristics of the matched pairs used for data analyses.

**Table 1:** Characteristics of matched pairs.

Subject Pair		EGA (weeks)	BW (g)	DOL Enteral Feed Initiation	DOL 1^st^ sample	Gender	Feed Type	DOL NEC
1	NEC	24	700	3	23	F	MM	50
	Control	25	670	5	24	F	MM	—
2	NEC	31	795	7	15	M	MM	32
	Control	29	940	4	14	F	MM	—
3	NEC	26	600	2	12	F	DM	31
	Control	24	590	5	12	F	MM	—
4	NEC	27	1050	3	4	F	MM	20
	Control	26	1100	3	6	M	MM	—
5	NEC	30	1605	8	13	F	MM + F	21
	Control	31	1585	6	10	M	MM + F	—
6	NEC	27	810	2	9	F	MM	17
	Control	27	940	2	9	M	MM	—
7	NEC	24	730	9	14	M	MM	27
	Control	25	640	8	13	F	MM + F	—
8	NEC	25	790	4	8	F	MM + DM	25
	Control	26	875	4	7	F	DM	—
9	NEC	27	925	6	10	F	MM	22
	Control	27	990	6	9	M	MM	—

EGA, estimated gestational age; BW, birth weight; DOL, day of life; DOL NEC, day of life of NEC diagnosis; MM, maternal milk; DM, donor milk; F, formula. EGA, BW, DOL enteral feed initiation and DOL 1^st^ sample were parameters used for matching.

### BA composition

**Figure 1:**
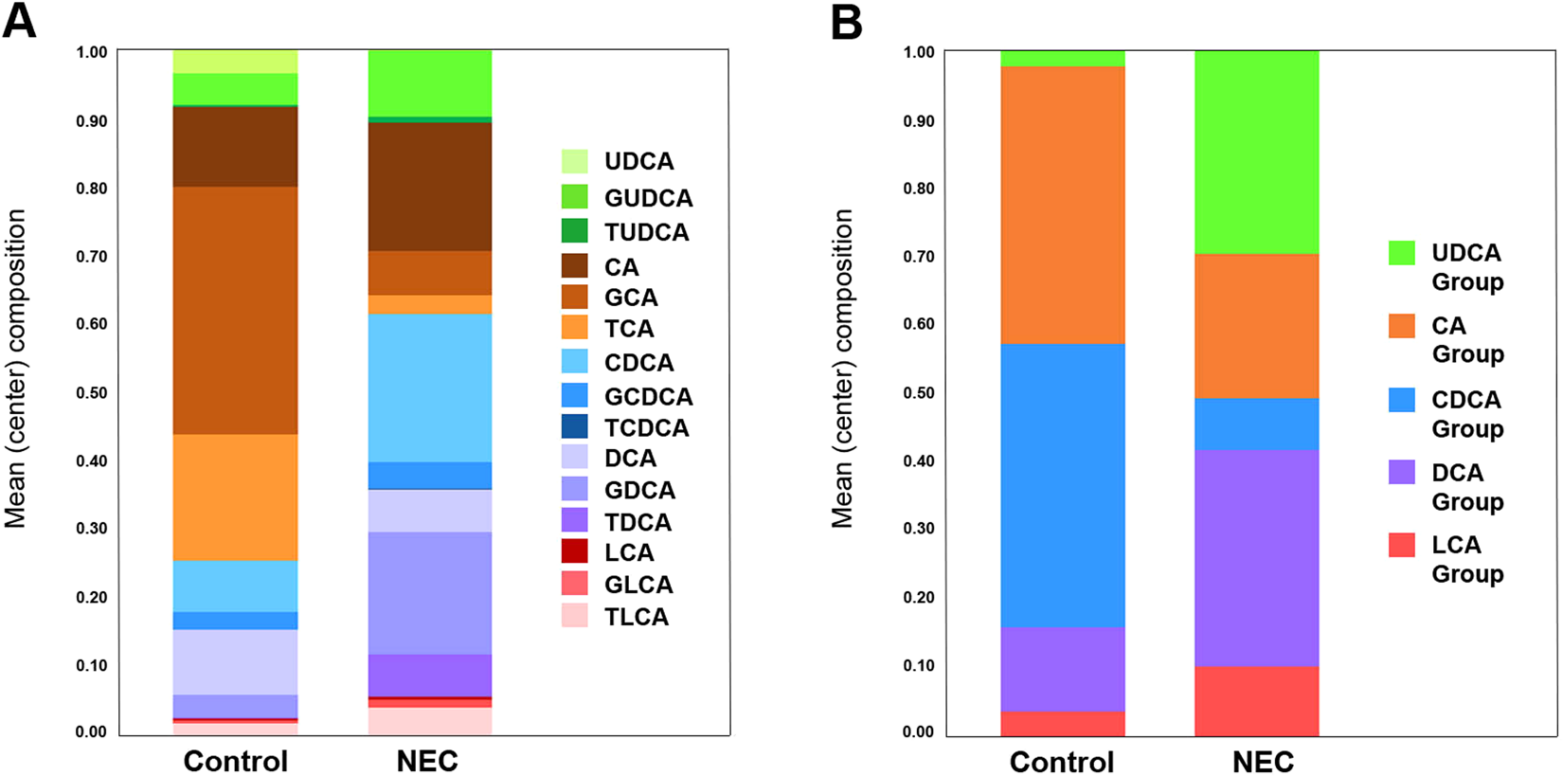
Mean (center) composition of individual (**A**) and grouped BAs (**B**) in control versus NEC. Grouped BAs include the unconjugated and conjugated species of UDCA, CA, CDCA, DCA and LCA. BAs are shown from top to bottom from least to most hydrophobic.

UDCA, CA, GCA, TCA, CDCA, GCDCA, TCDCA and TDCA comprised the main components of fecal BAs in the study population. Mean composition of the 15 individual bile acid species and of the five grouped species (all UDCA, CA, CDCA, DCA, and LCA) for NEC and Control subjects are shown in Fig. 1A, B, respectively. No statistically significant difference in overall composition was detected with either the 15 individual bile acids (*p*-value = 0.1133) or with the five bile acid groups (*p*-value = 0.0742).

### BA quantity

**Figure 2:**
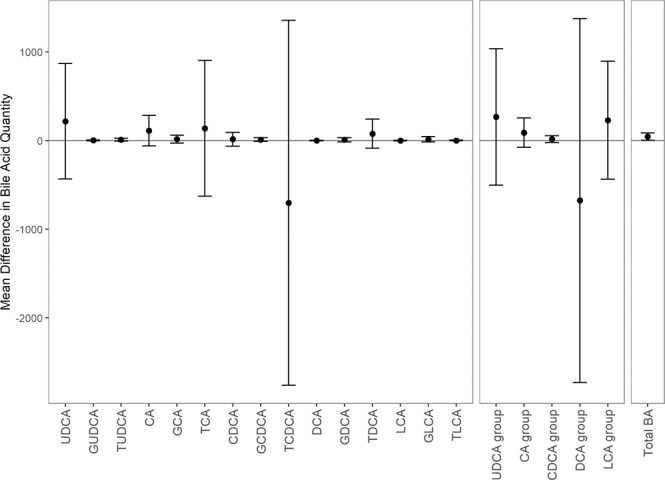
Mean difference in quantity of each bile acid between control and NEC pairs. Error bars represent ± 1 standard deviation as measured over the nine paired differences.

The mean difference between NEC and control subjects in quantity of total BA, as well as of each individual BA species and of the five BA groups, is shown in Fig. 2. After False Discovery Rate adjustment, no statistically significant difference was detected in any of the 21 comparisons (*p*-values all > 0.20).

### BA variation

**Figure 3:**
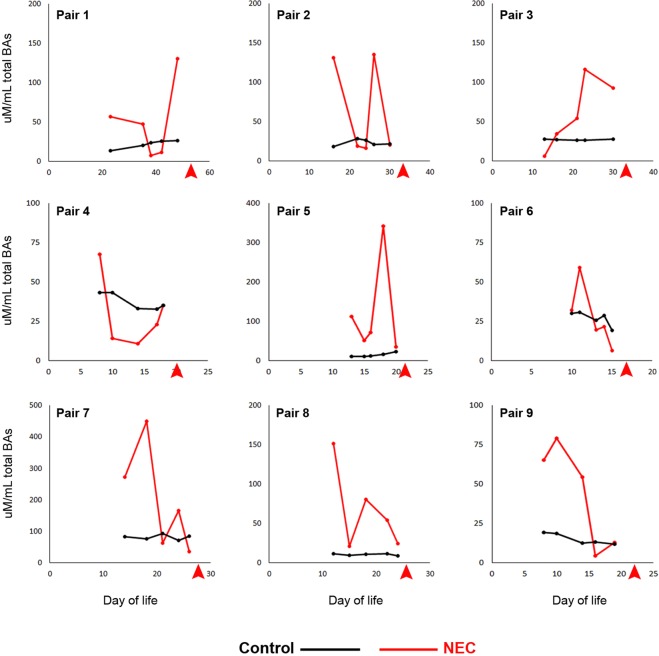
Total BA levels for each sample in each subject pair. Red arrows indicate day of life of NEC diagnosis.

Within pairs, the NEC subjects consistently had higher CV of total BA than the paired control, with the NEC subject having at least a 3-fold higher CV of total BA. The mean CV of total BA for NEC subjects was more than 4-times that of the Controls (Table 2). Notably, this variation is apparent well before NEC diagnosis (Fig. 3) and there was there was no overlap between the two groups: the smallest observed CV in the NEC subjects (0.5252) was larger than the largest CV of the control Subjects (0.3399, Table 2). The mean CV for each of the 15 individual BAs and for the five BA groups are shown in Tables 3 and 4, respectively. After adjusting for False Discovery Rate with these 21 comparisons, we detected a statistically significant difference in the mean CV of total BAs (p-value = 0.000012, Table 2), but not in any of the 15 individual BA species (Table 3) nor in any of the five BA groups (Table 4).

**Table 2:** Coefficient of variation (CV) for total BAs.

Subject Pair	Control CV	NEC CV
1	0.2348	0.9711
2	0.1824	0.9692
3	0.0264	0.7257
4	0.1411	0.7578
5	0.3399	1.0222
6	0.1740	0.5252
7	0.1019	0.8589
8	0.1146	0.8034
9	0.2331	0.7588
Mean CV (SD)*	**0.1720 (0.0911)**	0.8214 (0.1546)*

The CV for total BAs over the five samples was calculated for each subject and a paired t-test was conducted on the 9 pairs of CVs. *P = 0.000012 (adjusted for 21 multicomparisons).

**Table 3:** Coefficient of variation (CV) for individual bile acids.

	UDCA	GUDCA	TUDCA	CA	GCA	TCA	CDCA	GCDCA	TCDCA	DCA	GDCA	TDCA	LCA	GLCA	TLCA
Control	1.25 (0.64)	0.89 (0.48)	0.35 (0.70)	0.74 (0.39)	0.75 (0.40)	0.99 (0.57)	0.65 (0.46)	0.89 (0.544)	0.526 (0.57)	0.90 (0.50)	0.58 (0.25)	0.92 (0.59)	0.25 (0.74)	0.94 (0.90)	0.64 (0.65)
NEC	1.30 (0.58)	0.90 (0.47)	1.090 (0.801)	1.22 (0.52)	1.25 (0.68)	1.29 (0.44)	1.30 (0.28)	1.32 (0.61)	0.93 (0.81)	0.72 (0.49)	1.21 (0.66)	1.33 (0.74)	0.18 (0.53)	1.41 (0.80)	1.25 (0.80)
P value	0.92	0.99	0.17	0.18	0.16	0.23	0.09	0.29	0.39	0.47	0.16	0.29	0.92	0.38	0.18

Data shown as mean CV (SD). The CV of each bile acid over the five samples was calculated for each subject and a paired t-test was conducted on the nine pairs of CVs. P values adjusted for 21 multicomparisons.

**Table 4:** Coefficient of variation (CV) for BA groups.

	UDCA Group	CA Group	CDCA Group	DCA Group	LCA Group
Control	0.749 (0.424)	0.598 (0.257)	0.759 (0.466)	0.770 (0.444)	0.782 (0.728)
NEC	1.168 (0.486)	1.042 (0.499)	1.123 (0.366)	1.122 (0.584)	1.495 (0.620)
P value	0.173	0.175	0.229	0.207	0.161

UDCA group: UDCA, GUDCA, TUDCA. CA group: CA, GCA, TCA. CDCA group: CDCA, GCDCA, TCDCA. DCA group: DCA, GDCA, TDCA. LCA group: LCA, GLCA, TLCA. Data shown as mean CV (SD). The CV of each bile acid over the five samples was calculated for each subject and a paired t-test was conducted on the nine pairs of CVs. P values adjusted for 21 multicomparisons.

## Discussion

Despite advances in neonatal practice, NEC remains the most common GI emergency of premature infants. Immunologic^49–53^ and microbial initiators^37,41,54–57^ have been examined, but the pathophysiology of NEC has yet to be clearly elucidated and there are no predictive tests available. Studies in animal models of NEC strongly suggest accumulation of BAs play a significant role in disease development^18–21^. We found no statistically significant differences between control and NEC for any mean individual BA species, nor did we find statistically significant differences in mean total BAs. However, the mean CV for total BAs was statistically significantly higher in premature infants that develop NEC compared to matched controls. Importantly, variability occurred throughout the NEC subject’s measured samples and prior to NEC diagnosis. There was also no overlap in the CVs of total BAs, enhancing its potential as a prognostic measure.

These data show profound differences between magnitude of fluctuations of total fecal BAs between premature infants that develop NEC and those that do not. This suggests neonatal ileum may be especially sensitive to pulsatile exposure to BAs. In addition, fluctuation of total BA levels appears to be a better predictor of NEC development than mean overall BA levels. While exposure to varying levels of hydrophobic BAs has been shown to increase proliferation of cells in esophageal reflux and Barrett’s esophagus^58–60^, cytotoxic effects of intermittent exposure to BAs in neonatal intestine has not been published. While previous research reported a statistically significant increase in total unconjugated BAs in NEC patients compared to controls^48^, we did not observe a statistically significant increase in mean BA quantity for any individual BA, BA group, or total BAs. However, our study differed from theirs in that we used matched pairs, analyzed more samples per subject, looked at slightly different outcomes, and had lower power due to a smaller sample size and correcting for multiple comparisons (that is, we required a lower p-value for “statistical significance”). While we observed a trend toward higher BAs in NEC, extreme variation between an individual NEC patient’s samples prevented reaching statistical significance in this relatively modest sample size. However, this variability is in itself, a potential predictor of NEC development.

Although no single pathogen has been definitively associated with NEC^55–57^, the intestinal microbiome has a profound effect on the composition of intestinal BAs. Conversion of BAs from primary to secondary forms requires bacterial dehydroxylation by species of *Clostridium* and *Eubacterium* in the intestine^61–66^ and *Clostridia* are abundant in the feces of infants with NEC^14,67,68^. Further, deconjugation of BAs depends on separate bacterial conversions^69^. While postnatal antibiotic use has been suggested as an additional risk factor for NEC^70,71^, antibiotic use in all subjects from both groups were similar (treatment within the first seven days of life). Further investigation of changes in the microbiome during development of NEC, specifically with regard to bacteria capable of BA transformations, is ongoing but exceeds the scope of this work.

Whether fluctuations in total BAs leads to NEC or is a consequence of other pathophysiologic changes that occur prior to NEC has yet to be determined. The intestinal epithelial barrier is inherently leaky in any neonate but tight junctions (TJs), an integral component of intestinal barrier integrity, are altered in NEC^72–77^ and disrupted after continuous exposure to hydrophobic BAs^78–80^. Pulsatile exposure to BAs may produce more profound changes in TJs and barrier function that allows aberrant movement of proinflammatory mediators out of the intestinal epithelium as well as bacterial products into cells or systemic circulation if epithelial integrity is compromised.

The apical sodium-dependent bile acid transporter (ASBT) is the primary transporter of BAs across the apical membrane of enterocytes^81–83^. To prevent toxicity from intracellular accumulation of BAs, BAs repress ASBT via interaction with the farnesoid X receptor (FXR)^84,85^. However, we determined that surface expression of ileal ASBT is increased in NEC, leads to accumulation of intra-enterocyte BAs, and increases the incidence and severity of experimental NEC^18,19^. Further, ASBT is also increased on the surface of ileal surgical samples from infants diagnosed with NEC^19^. Consequently, changes in ASBT regulation after exposure to fluctuating BA levels must be examined as a potential mechanism to explain how pulsatile exposure to BAs may be more cytotoxic than continuous exposure.

The statistical power of our analyses was limited by two key factors. First, there were large standard deviations in the quantities of several BAs, some of which were attributed to individual subjects (e.g., elevated levels UDCA for a single subject in the NEC group and TCDCA for a single subject in the control group). This affected both the analysis of mean BA quantity as well as of the overall composition. Second, the sample size was limited to only nine pairs of subjects. This is, in part, due to the limited number of subjects that develop NEC, but also to the issue of finding appropriate controls (both subject pairs as well as sample pairs) for comparison. The variability in stooling frequency coupled with inability to control stooling frequency further contributed to our limited sample size. It is also possible that variation in stooling frequency affected the measured response (the amount, either absolute or proportional, of various BAs). Sample collection has begun at two additional NICUs which will increase the number of NEC patients, allowing for analysis of matched pairs in a ratio of 1:2 (NEC:Controls) and compaction of tolerance for EGA in future studies. Parameters used to establish the matched pairs did not include gender or type of enteral feeding. Gender is not considered a risk factor for NEC^86^, BAs do not differ significantly by gender^87,88^ and the type of enteral feeding was quite similar between matched pairs (Table 1). The only pair with significant differences in diet—Pair 7—the control subject was the one given formula (and only for four feedings), which is considered a risk factor for NEC^12^. Thus, we considered the differences in gender and feed type unlikely to have influenced our results.

In conclusion, variation in total BAs occurred well in advance of NEC diagnosis with no overlap in CV between the two groups (all nine NEC subjects had CV of total BA greater than that of any of the control subjects). While larger clinical studies must be performed, these data suggest the CV of total BAs is a biomarker of NEC and has the potential to be developed as the first predictive test for this devastating disorder.

## Methods

### Study participants

Following approval by the University of Arizona Institutional Review Board, premature infants were enrolled prospectively via informed, written parental consent at Banner University Medical Center Tucson. All research was performed in accordance with relevant regulations. The inclusion criteria—birth weight (BW) ≤ 1800 g, estimated gestational age (EGA) ≤ 32 weeks, and < 30 days old prior to initiation of enteral feeding—were chosen because NEC occurs almost exclusively in premature infants, the most premature infants are more likely to develop disease, and most cases occur after the initiation of enteral feeding^89–91^. Exclusion criteria included conditions not related to prematurity including blood-culture positive sepsis or genetic syndromes and were based on eliminating subjects that could develop NEC-like syndromes due to other confounding problems not related to the most common risk factors for NEC. Definitions of NEC diagnosis and time of diagnosis were defined as any subject with Bell’s Stage ≥ II (modified Bell’s staging criteria) and radiographic evidence of NEC, respectively.

### Sample collection and analysis

Post-meconium fecal samples were collected from the diaper for up to four weeks after initiation of enteral feeding. Samples were placed in sterile microtubes, frozen in the NICU at −20 °C and transported to the laboratory weekly where they were then stored at −80 °C until processing. Analysis of various freezing protocols prior to actual sample collection determined this freezing protocol did not alter BA levels or composition (data not shown). For analysis, samples were thawed, weighed, and mixed with an equal volume of nanopure water. After homogenization, samples were centrifuged to separate fecal water from the solids and the fecal water was frozen at −80 until BAs were assayed^28,92^. BA composition was determined using the ThermoFinnigan TSQ Quantum Ultra Mass Spectrometer with High Performance Liquid Chromatography System (Arizona Cancer Center Analytical Core Shared Service). Fifteen separate BA species—UDCA, CA, DCA, CDCA, LCA, along with their glycol and tauro conjugates—were evaluated^93^. The LC-MS/MS method utilized to determine individual BA species does not measure hyocholic acid (HCA) or hyodeoxycholic (HDCA), which can contribute significantly to total BAs in infants^92^. Therefore, to determine total BA levels, we utilized a commercially available kit that measures all BAs via an enzymatic cycling method with spectrophotometric readout^18,19^. Total BAs were calculated using the Diazyme Total Bile Acids Assay Kit (Diazyme Laboratories, Poway, CA, USA).

### Statistical analyses

Due to the nested-nature of samples, pairing was accomplished via a two-stage process: 1) appropriate control subjects were first paired to each NEC subject, then 2) paired samples within those subjects were selected. A control subject was considered an appropriate match for a NEC subject if the following four tolerances were met: EGA within 2.5 weeks, BW within 150 g, initiation of enteral feeding within 3.5 days, and the day of life of the first sample was within 3.5 days. Once a set of potential control pairs was identified using these tolerances for each NEC subject, the single control pair was selected for each NEC subject as follows. Because a single control subject could be a potential match for more than one NEC subject, we first ensured that all NEC subjects with potential controls were paired with a control (if a control was a potential pair with a NEC subject that had several potential control-subjects and also was a potential pair with a NEC subject with only one potential control, it would be paired with the NEC subject with only one potential control). Of the remaining controls that were potential matches to a NEC subject, we looked for pairs that had at least five samples on the same day (days from birth) as the control. Our final dataset was composed of nine NEC subject pairs, each with five paired samples. All NEC subject samples measured were collected prior to NEC diagnosis and all subjects received antibiotics in the first week of life.

To assess whether there was a difference in BA composition between NEC and control subjects, we conducted two separate analyses, one using each of the fifteen BAs analyzed and another where the BAs were combined into groups: UDCA group (UDCA + GUDCA + TUDCA), CA group (CA + GCA + TCA), CDCA group (CDCA + GCDCA + TCDCA), DCA group (DCA + GDCA + TDCA), and LCA group (LCA + GLCA + TLCA). Prior to analysis, composition data was additive log-ratio (ALR) transformed^94^. The ALR-transformation takes the natural-log of the proportion for each of the D−1 components divided by the proportion of the Dth component. For the ALR-transformation, the Dth (denominator) component was CA (for the analysis of the 15 BAs) and the CA group (for the analysis of the five BA groups). Due to the complex, hierarchically paired structure of the data, a standard MANOVA was not appropriate. Instead, a permutation test was performed. In order to preserve the nested and paired structure of the data, for each permutation we only randomly permuted which subject within a pair was given the label “NEC” vs. “control”. Subject and sample pairs were maintained. As there were only nine pairs, there were only 2^9^ = 512 permutations. The test statistic used in the permutation test was the Pillai’s Trace for diagnosis (NEC vs. control) from a MANOVA with fixed effects for Sample Pair^95^.

To visualize a representative composition of BAs and BA groups for NEC vs. control subjects, the compositional mean was calculated for each diagnosis group by determining the geometric means of the proportion of each BA over all 45 samples for a diagnosis group and standardizing to 1 by dividing by the sum of those geometric means^96^.

In addition to examining overall composition, we also examined whether there was a difference between control and NEC subjects in the mean quantity and in the mean coefficient of variation (CV) of the quantity of total BA as well as in each of the 15 BAs and each of the five BA groups. For these analyses we, again, used the same nine pairs of subjects and the same five pairs of samples within each pair of subjects. For each BA, the difference in the quantity of that BA between sample pairs was calculated. We then took the average of these five differences within each Subject Pair. A 1-sample *t*-test was used to test whether the mean difference in quantity was equal to zero or not. The CV of each BA over the five samples was calculated for each subject and a paired *t*-test was conducted on the nine pairs of CVs. Because testing for differences in mean quantity and differences in mean CV each involved a total of 21 tests, we adjusted p-values for 21 multiple comparisons using the false discovery rate (FDR) method of Benjamini and Hochberg^97^.

## Data Availability

Data are available from the authors upon reasonable request.
